# Supplementary material for: Comparison of Non-Contrast CT vs. Contrast-Enhanced CT with Both Intravenous and Rectal Contrast Application for Diagnosis of Acute Colonic Diverticulitis: A Multireader, Retrospective Single-Center Study
Source: Diagnostics (Basel). 2024 Dec 26;15(1):29. doi: 10.3390/diagnostics15010029 (PMC11719699; doi:10.3390/diagnostics15010029)
Supplement: Supplementary file 1 [file diagnostics-15-00029-s001.zip › diagnostics-3385857-supplementary.pdf]

Comparison of Non-Contrast CT vs. Contrast-Enhanced CT with Both Intravenous and Rectal Contrast Application for Diagnosis of Acute Colonic Diverticulitis: A Multireader, Retrospective Single-Center Study

Supplementary Material

Additional information on statistical methods.

Continuous variables are presented as the mean±standard deviation (mean±SD), median with interquartile range (IQR) (median (Q1, Q3)). Categorical data are presented as absolute and relative frequencies. Continuous variables were compared between groups using Welch's two-sample t-test while the confidence intervals (CI) for the difference in means were derived from the t-distribution. Categorical variables were compared with Fischer´s exact test and Pearson's chi^2 test and the difference in proportions were derived from a normal ("Wald") approximation.

Diagnostic performance was analysed by calculating sensitivity, specificity and accuracy for the non-contrast and for the contrast-enhanced CT group, as well as the difference of the groups using the methods of Saeki and Tango [23]. Associated confidence intervals were calculated by means of linear regression models including the group variable (non-contrast vs. contrast-enhanced CT) only, as the random effects for reader were estimated to be zero in all models. The dependent variables in the regression models were coded as 1: correct decision and 0: incorrect decision for accuracy, 1: true positive and 0: false negative for sensitivity and 1: true negative and 0: false positive for specificity, respectively. The proposed methods are for comparing the sensitivities, specificities and accuracies of two diagnostic procedures and whereby all images are read by all readers. Furthermore, it was evaluated whether the expertise of a reader influences the accuracy, sensitivity or specificity by means of linear regression models adjusting for the variables group and expert (expert vs. non-expert reader).

Considerations regarding the extra time expenditure for CT scans with both rectal and intravenous contrast compared to non-contrast CT scans:

Compared to a non-contrast CT scan, a CT scan with contrast enhancement is associated with a higher complexity of patient and equipment preparation, and of the CT scanning itself. The additional time needed for an informed consent for a contrast-enhanced CT scan compared to a non-contrast CT scan is estimated to be about 2 minutes [30]. A conservative estimation for insertion and testing of a peripheral intravenous catheter as well as connection to and preparation of the contrast agent injector is 5 minutes [31] (this time is reduced if the patients arrives in the radiology department with an appropriate intravenous catheter). In our experience, the preparation and dilution of the rectal contrast agent, placement of the rectal catheter, and administration of the rectal contrast agent takes about 5 minutes. The scan delay for the portal venous phase after the start of intravenous contrast application prolongs the examination by about 1.5 minutes. We estimate the medium additional cleaning time of the CT table after rectal contrast application to be 1.5 minutes, not taking into account the contact time of an additionally used disinfectant (how long the disinfectant must stay wet on the CT table to kill pathogens) [32]. These estimations result in an additional time expenditure for contrast-enhanced CT scans compared to non-contrast CT scans of up to 15 minutes. Therefore, urgent non-contrast CT scans can be more easily performed in a short time window between two scheduled CT scans than contrast-enhanced CT scans. In one study, this led to a prolongation of the time to scan completion for contrast-enhanced CT scans of the abdomen/ pelvis by more than one hour [33].

Supplementary Table S1. Classification of diverticulitis cases according to the modified Hinchey classification (reference standard).

Stage	Definition	Number of cases
Ia	Confined pericolic inflammation without perforation or abscess	85
Ib	Contained perforation and/or confined pericolic abscess	32
II	Pelvic, distant intraabdominal, or retroperitoneal abscess	13
III/IV	Free perforation with generalized purulent (III) or fecal (IV) peritonitis	6

Stages III and IV were pooled since they can be impossible to differentiate by imaging alone. There were no cases with leakage of rectal contrast material into adjacent hollow organs (proof of a fistula) or with pylephlebitis (septic thrombosis of the portal vein) secondary to colonic diverticulitis.

Supplementary Table S2. Size, localisation and reader rating of abscesses (pooled).

% of all abscesses		reference standard	**identification** of abscesses **by readers** (pooled)					localisation
			identified in n.c.	identified in c.e.	identified only in n.c.	identified only in c.e.	not identified	
**all** abscesses		100	53	69	8	24	23	
**diverticulitis** associated		89	49	65	7	22	18	
	Hinchey Ib	43	14	29	1	16	5	sigmoid colon
			1	-	1	-	4	descending colon
			-	1	-	1	2	ascending colon
	Hinchey II	35	20	20	3	3	3	interloop/deep pelvic
			2	3	-	1	-	retroperitoneal
			4	4	1	1	1	ovary
	Hinchey III/IV	11	2	2	-	-	1	sigmoid colon
			4	5	-	1	1	descending colon
			2	1	1	-	1	ovary
**non-diverticulitis** associated		11	2	2	1	1	-	sigmoid colon
			1	2	-	1	1	periappendiceal
			-	1	-	1	2	ovary
			1	-	1	-	2	inguinal
**median diameter** [mm] (IQR)		45 (31)	55 (37)	47 (30)	41 (32)	33 (26)	29 (23)	

This table includes all abscesses (n = 37 according to the reference standard), both diverticulitis-associated and non-diverticulitis associated. The left part of the table shows the prevalence and median diameter with interquartile range of the abscesses according to the reference standard. The right part of the table shows the prevalence and size according to the reader ratings: identified in non-contrast (n.c.) CT, identified in contrast-enhanced (c.e.) CT, identified only in non-contrast CT (but missed in contrast-enhanced CT), identified only in contrast-enhanced CT (but missed in non-contrast CT) or not identified in either non-contrast or c.e CT (as % of 37 abscesses x 5 readers =185 readings, listed according to their localisation and Hinchey type).

The diameter of abscesses reported by readers only in contrast-enhanced CT did not differ significantly from those abscesses reported neither in non-contrast nor in contrast-enhanced CT (p-value of the Student`s t-test: 0.321).

The abscesses identified only in contrast-enhanced CT were rather small and most frequently Hinchey type Ib on the sigmoid colon.

Supplementary Table S3. Differences between expert vs. non-expert readers.

experts' advantage	**diverticulitis**		**perforation**		**perforation: contained**		**perforation: free**		**abscess**	
		95% CI		95% CI		95% CI		95% CI		95% CI
accuracy	0.005	(-0.0208, 0.0297)	0.009	(-0.0200, 0.0379)	0.021	(-0.0095, 0.0518)	0.014	(0.0021, 0.0256)	0.015	(-0.0137, 0.0430)
sensitivity	0.017	(-0.0129, 0.0473)	-0.049	(-0.1237, 0.0260)	-0.012	(-0.1010, 0.0767)	0.042	(-0.1045, 0.1878)	-0.032	(-0.1320, 0.0689)
specificity	-0.021	(-0.0664, 0.0253)	0.032	(0.0077, 0.0558)	0.031	(0.0070, 0.0556)	0.012	(0.0028, 0.0220)	0.025	(0.0023, 0.0474)

Given are the differences of accuracies, sensitivities and specificities with their 95% confidence intervals (CI) for summated non-contrast and contrast-enhanced readings of board-certified radiologists (expert; R1,2) vs. trainee readers (non-expert; R3-5) for the diagnosis of colonic diverticulitis, abscess (dichotomous: present/absent) and perforation (contained/free).

A significant advantage of expert vs. non-expert readers could be observed for the specificity of perforation (overall, contained and free), abscess and for the accuracy of free perforation. Confidence intervals for all other endpoints and for specificity of sigmoid diverticulitis included the 0.

Supplementary Table S4. Intraluminal contrast filling and extralumination.

	**reference st.**		**R1**		**R2**		**R3**		**R4**		**R5**	
	reached	extralum.	reached	extralum.	reached	extralum.	reached	extralum.	reached	extralum.	reached	extralum.
n	50	8	47	1	45	2	50	4	45	13	50	3
% of all cases	24	4	23	0	22	1	24	2	22	6	24	1
% of reached		16		2		4		8		29		6

Given are the number and prevalence of cases with sufficient intraluminal contrast at the site of the perforation and the percentage of detected contrast extralumination (extralum.) according to the reference standard (st.) and the individual readers.

Supplementary Table S5. Association of contrast application and successful detection of alternative diagnoses.

	Fisher´s exact test		chi^2 test	
	Phi	p-value	chi^2	p-value
R1	-0.08	0.619	0.25	0.617
R2	-0.03	1.000	0	1.000
R3	-0.15	0.233	1.42	0.233
R4	-0.21	0.074	3.15	0.076
R5	-0.16	0.189	1.72	0.190
all readers	-0.13	0.008	6.97	0.008
certified (R1+R2)	-0.06	0.559	0.34	0.560
trainees (R3+R4+R5)	-0.17	0.007	7.27	0.007

Association of contrast application and successful detection of alternative diagnoses examined with Fischer´s exact test and chi^2 test for each individual reader (R1-5), all readers, board certified radiologists (experts; R1,2) and radiology trainees (non-experts; R3-5).

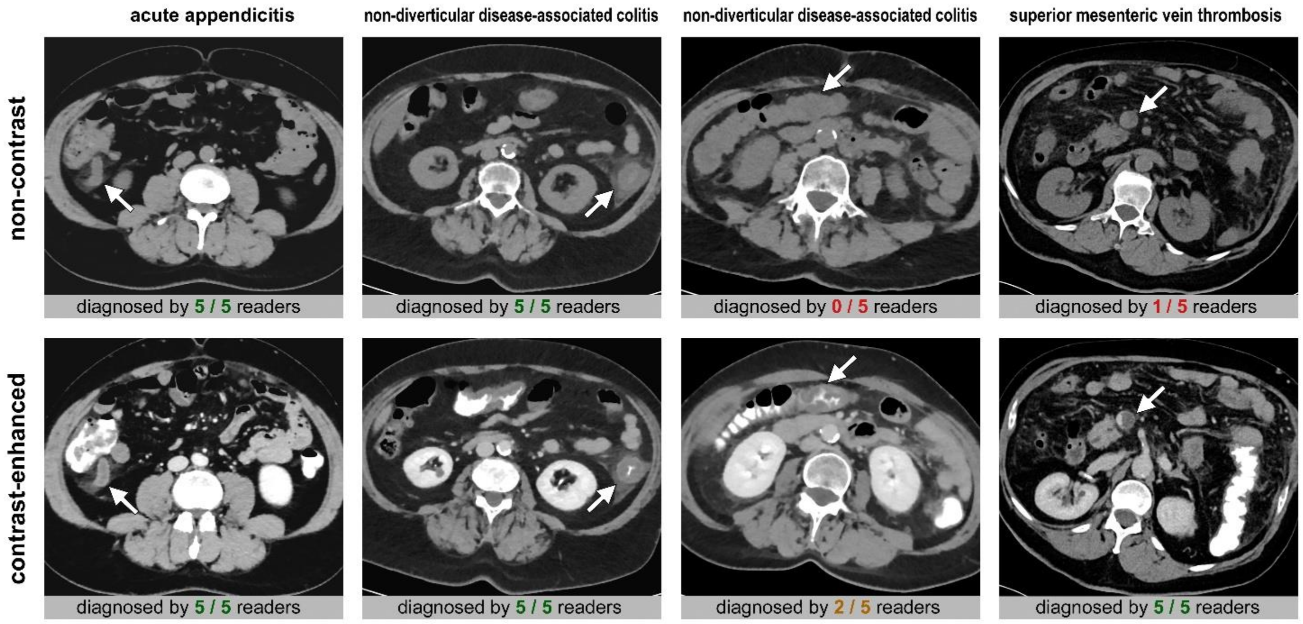

Supplementary Figure S1. Example images of acute abdominal conditions other than diverticulitis. The upper row shows representative non-contrast CT images, the lower row shows the corresponding contrast-enhanced CT images. The number of accurate readings is given below each CT image. Left and middle left: appendicitis (arrow) and non-diverticulis-associated colitis with perifocal oedema of the mesenteric fat, correctly diagnosed in most cases both in non-contrast CT and contrast-enhanced CT. Middle right and right: Non-diverticulitis-associated colitis (with only minimal surrounding fat stranding, arrow) and thrombosis of the mesenteric vein (arrow) missed by the majority of readers in non-contrast CT.
